# Supplementary material for: The Combination of Citrus Rootstock and Scion Cultivar Influences Trioza erytreae (Hemiptera: Triozidae) Survival, Preference Choice and Oviposition
Source: Insects. 2024 May 16;15(5):363. doi: 10.3390/insects15050363 (PMC11122159; doi:10.3390/insects15050363)
Supplement: Supplementary file 1 [file insects-15-00363-s001.zip › insects-2960820-supplementary.pdf]

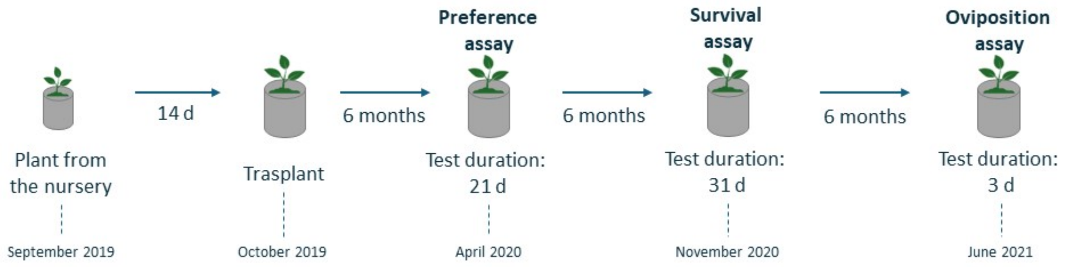

Figure S1. Scheme of the use of each citrus combination in chronological order of experiments with *T. erytreae*.

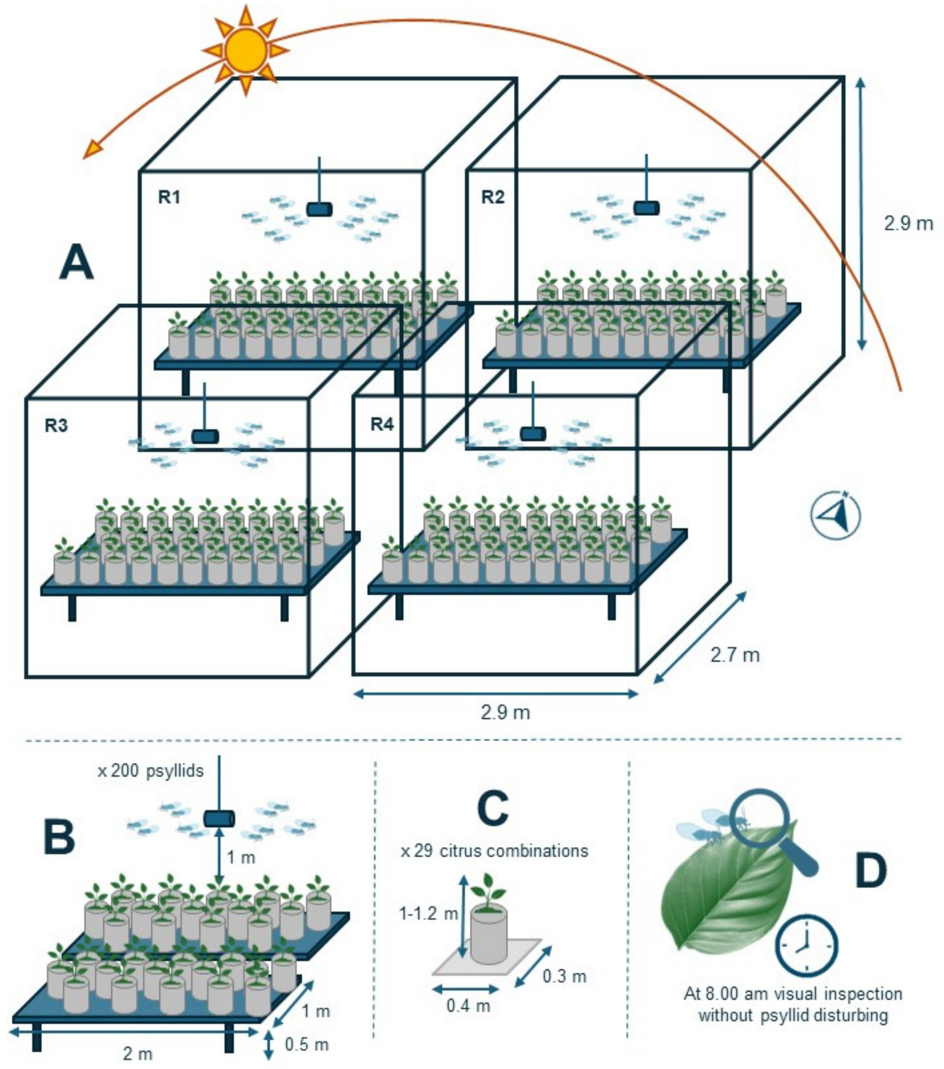

Figure S2. Scheme of *T. erytreae* settlement preference assay. A, localization of replicates (4 walk-in entomological cages) used in this experiment; B, details of psyllids release; C, dimension and occupied area per plant; and D, detail of psyllid monitoring.

Table S1. Kaplan-Meier estimate for the 'Ortanique' cultivar.

Rootstock *	Time (days)	Dead (n)	Alive (n)	Estimated probability		Probability of survivors at the end of time
				Survival	Death	
CC	1	7	25	0.964	0.036	0.964
	3	13	19	0.885	0.115	0.853
	7	15	17	0.782	0.218	0.667
	17	29	3	0.545	0.455	0.364
	24	32	0	0.273	0.727	0.099
	31	32	0	0.000	1.000	0.000
CM	1	6	26	0.969	0.031	0.969
	3	9	23	0.914	0.086	0.886
	7	11	21	0.836	0.164	0.740
	17	26	6	0.609	0.391	0.451
	24	31	1	0.314	0.686	0.142
	31	31	1	0.010	0.990	0.001
FA5	1	4	28	0.979	0.021	0.979
	3	8	24	0.930	0.070	0.911
	7	10	22	0.858	0.142	0.781
	17	26	6	0.625	0.375	0.488
	24	31	1	0.322	0.678	0.157
	31	31	1	0.010	0.990	0.002
FA517	1	13	19	0.932	0.068	0.932
	3	15	17	0.845	0.155	0.788
	7	20	12	0.713	0.287	0.562
	17	29	3	0.498	0.502	0.279
	24	32	0	0.249	0.751	0.069
	31	32	0	0.000	1.000	0.000
FD	1	9	23	0.953	0.047	0.953
	3	12	20	0.882	0.118	0.840
	7	13	19	0.792	0.208	0.666
	17	23	9	0.602	0.398	0.401
	24	27	5	0.348	0.652	0.140
	31	28	4	0.044	0.956	0.006

*Rootstock: FD, Flying Dragon; CC, Carrizo citrange; FA5, Forner-Alcaide no. 5; FA517, Forner-Alcaide no. 517; CM, *Citrus macrophylla*

Table S2. Kaplan-Meier estimate for the cultivars: ‘Clemenules’, ‘Navelina’, ‘Valencia Late’ and ‘Fino 49’.

Cultivars	Root-stock*	Time (days)	Dead (n)	Alive (n)	Estimated probability		Probability of survivors at the end of time
					Survival	Death	
‘Clemenules'	CC	1	9	23	0.953	0.047	0.953
		3	9	23	0.900	0.100	0.857
		7	9	23	0.836	0.164	0.717
		17	27	5	0.601	0.399	0.431
		24	32	0	0.301	0.699	0.130
		31	32	0	0.000	1.000	0.000
	CM	1	10	22	0.948	0.052	0.948
		3	11	21	0.883	0.117	0.837
		7	13	19	0.793	0.207	0.664
		17	31	1	0.537	0.463	0.356
		24	32	0	0.268	0.732	0.096
		31	32	0	0.000	1.000	0.000
‘Navelina'	CC	1	8	24	0.958	0.042	0.958
		3	8	24	0.910	0.090	0.872
		7	8	24	0.854	0.146	0.745
		17	27	5	0.613	0.387	0.457
		24	31	1	0.316	0.684	0.145
		31	32	0	0.000	1.000	0.000
	CM	1	7	25	0.964	0.036	0.964
		3	10	22	0.903	0.097	0.870
		7	11	21	0.826	0.174	0.719
		17	20	12	0.654	0.346	0.470
		24	23	9	0.419	0.581	0.197
		31	24	8	0.105	0.895	0.021
‘Valencia Late'	CC	1	14	18	0.927	0.073	0.927
		3	14	18	0.846	0.154	0.784
		7	21	11	0.707	0.293	0.555
		17	32	0	0.471	0.529	0.261
		24	32	0	0.236	0.764	0.062
		31	32	0	0.000	1.000	0.000
	CM	1	7	25	0.964	0.036	0.964
		3	8	24	0.915	0.085	0.882
		7	10	22	0.844	0.156	0.744
		17	27	5	0.607	0.393	0.451
		24	30	2	0.322	0.678	0.145
		31	30	2	0.020	0.980	0.003
‘Fino 49'	CC	1	6	26	0.969	0.031	0.969
		3	10	22	0.908	0.092	0.880
		7	12	20	0.823	0.177	0.724
		17	24	8	0.617	0.383	0.447
		24	31	1	0.318	0.682	0.142
		31	31	1	0.010	0.990	0.001
	CM	1	9	23	0.953	0.047	0.953
		3	11	21	0.888	0.112	0.846
		7	15	17	0.784	0.216	0.663
		17	22	10	0.604	0.396	0.400
		24	27	5	0.349	0.651	0.140
		31	28	4	0.044	0.956	0.006

*Rootstock: CC, Carrizo citrange; CM, *Citrus macrophylla*.

Table S3. Number of individuals of *T. erytreae* (mean±SE) settled per citrus combination during six monitoring times (1, 3, 7, 14 and 21 days).

Scion cultivar	Rootstock*	Days from psyllids release				
		Day 1	Day 3	Day 7	Day 14	Day 21
'Clemenules'	CC	4.0±0.4	5.0±1.1	1.8±0.8	0.0±0.0	0.3±0.3
	CL	2.0±0.9	3.0±0.9	1.0±0.6	1.8±0.3	0.3±0.3
	CM	2.5±1.0	1.8±0.9	1.5±0.9	1.0±1.0	0.3±0.3
	FA5	1.8±0.5	1.8±0.9	1.8±1.0	1.0±0.4	0.5±0.5
	FA517	0.0±0.0	0.3±0.3	1.5±1.2	0.3±0.3	0.0±0.0
	FD	2.0±0.4	1.5±0.5	0.5±0.3	1.8±0.3	1.3±0.5
'Fino 49'	CC	0.3±0.3	0.3±0.3	0.3±0.3	0.5±0.3	0.0±0.0
	CM	2.5±0.9	2.3±0.9	1.3±0.8	0.3±0.3	0.5±0.5
	FA5	3.0±0.6	2.3±1.1	1.3±0.9	0.0±0.0	0.3±0.3
	FD	2.8±1.3	3.0±1.3	2.3±0.9	1.0±0.6	0.3±0.3
'Navelina'	CC	4.3±1.7	4.3±2.0	2.8±1.1	2.0±1.1	1.3±0.5
	CLE	2.8±0.6	3.0±0.7	2.3±1.6	1.3±0.9	0.3±0.3
	CM	2.3±0.6	2.0±0.7	0.5±0.5	1.3±0.8	1.0±1.0
	FA5	2.8±1.3	3.3±1.7	1.3±0.9	1.0±0.7	0.5±0.5
	FA517	1.3±0.6	2.3±1.7	1.8±1.0	2.0±0.8	0.3±0.3
'Ortanique'	CC	5.3±2.3	3.5±1.7	0.8±0.3	0.3±0.3	0.5±0.5
	CM	4.0±2.0	4.0±2.3	2.0±1.2	0.0±0.0	0.0±0.0
	FA5	2.3±0.8	0.8±0.5	1.8±0.8	1.0±0.7	0.8±0.8
	FA517	4.0±2.3	4.0±1.2	4.3±1.1	2.5±1.3	1.0±0.6
	FD	3.0±1.2	2.0±0.8	3.8±2.4	3.5±2.9	3.3±2.9
'Star Ruby'	CC	1.8±1.1	2.3±1.6	0.5±0.3	0.3±0.3	0.0±0.0
	CLE	3.3±2.9	2.0±1.7	0.8±0.5	0.5±0.5	1.0±0.7
	FA5	1.8±0.8	3.3±1.5	1.5±1.2	1.0±0.6	0.5±0.3
	FD	2.0±0.4	2.0±1.4	1.8±0.9	0.8±0.8	0.3±0.3
'Valencia Late'	CC	1.8±1.0	1.5±1.2	0.8±0.5	0.0±0.0	0.3±0.3
	CLE	1.5±1.5	1.0±0.7	1.8±0.9	1.0±0.7	0.3±0.3
	CM	2.8±1.8	2.5±1.9	0.3±0.3	0.8±0.5	0.0±0.0
	FA5	1.8±0.5	1.5±0.9	1.3±0.6	0.8±0.8	0.5±0.3
	FD	1.3±0.5	0.8±0.5	0.8±0.8	0.8±0.8	2.3±1.3

*Rootstock: FD: Flying Dragon; CL: Cleopatra mandarin; CC: Carrizo citrange; FA5: Forner-Alcaide no. 5; FA517: Forner-Alcaide no. 517; CM: Citrus macrophylla.
